# Bacteriophages Reduce Pathogenic *Escherichia coli* Counts in Mice Without Distorting Gut Microbiota

**DOI:** 10.3389/fmicb.2019.01984

**Published:** 2019-09-10

**Authors:** Upuli Dissanayake, Maria Ukhanova, Zachary Daniel Moye, Alexander Sulakvelidze, Volker Mai

**Affiliations:** ^1^Department of Epidemiology, College of Medicine, College of Public Health and Health Professions, University of Florida, Gainesville, FL, United States; ^2^Laboratory 300B, Department of Epidemiology, Emerging Pathogens Institute, University of Florida, Gainesville, FL, United States; ^3^Intralytix, Inc., Baltimore, MD, United States

**Keywords:** bacteriophage, phage, *E. coli*, foodborne disease, microbiome

## Abstract

We performed a study to (i) investigate efficacy of an *Escherichia coli/Salmonella* spp*./Listeria monocytogenes*-targeting bacteriophage cocktail (tentatively named F.O.P.) to reduce a human pathogenic *E. coli* strain O157:H7 in experimentally infected mice, and (ii) determine how bacteriophages impact the normal gut microbiota when compared with antibiotic therapy. A total of 85 mice were inoculated with *E. coli* O157:H7 strain Ec231 [nalidixic acid resistant (NalAc^R^)] via oral gavage, and were randomized into six groups separated into three categories: 1st category received PBS or No phage/No PBS (control), 2nd category received either F.O.P., F.O.P. at 1:10 dilution, or only the *E. coli* phage component of F.O.P. (EcoShield PX^TM^), and 3rd category received the antibiotic ampicillin. All therapies were administered twice daily for four consecutive days including before and after bacterial challenge; except ampicillin which was administered only before and after bacterial challenge on day 0. Fecal samples were collected at Days 0, 1, 2, 3, 5, and 10. Samples were homogenized and plated on LB plates supplemented with NalAc to determine viable Ec231 counts. Body weights were measured at every fecal sample collection point. qPCR was performed using specific *E. coli* O157:H7 primers to quantify the number of *E. coli* O157:H7 genome copies. Microbiota community profiles were analyzed using Denature Gradient Gel Electrophoresis (DGGE) and 16S rRNA sequencing. F.O.P. significantly (*P* < 0.05) reduced *E. coli* O157:H7 pathogen counts by 54%. Ampicillin therapy significantly (*P* < 0.05) reduced *E. coli* O157:H7 pathogen counts by 79%. Greater initial weight-loss occurred in mice treated with ampicillin (−5.44%) compared to other treatment groups. No notable changes in the gut microbiota profiles were observed for control and F.O.P. groups. In contrast, the antibiotic group displayed noticeable distortion of the gut microbiota composition, only partially returning to normal by Day 10. In conclusion, we found that F.O.P. administration was effective in reducing viable *E. coli* O157:H7 in infected mice with a similar efficacy to ampicillin therapy. However, the F.O.P. bacteriophage preparation had less impact on the gut microbiota compared to ampicillin.

## Introduction

Antibiotic resistance has become increasingly problematic over recent decades. With limited development of novel antibiotic drugs, antibiotic resistance poses an increasingly severe threat to the health of the general population. The main causes driving antibiotic resistance are thought to include their overuse in humans (including prophylactic use), excessive use in veterinary medicine, and ecological sources that include common farming practices (Lutter et al., 2005; Sengupta et al., 2013). Potentially beneficial bacteria that are not targeted but also sensitive to the antibiotic are also eliminated, leaving the host vulnerable (Stapleton and Taylor, 2002; Ventola, 2015). Between the plateau in development of new antibiotics, the damage caused to indigenous microbiota, and the rapid emergence of decreased sensitivity toward common antibiotics, health professionals have a need for alternative therapies to fill growing gaps in treatment options (Ashkenazi et al., 1991; Lushniak, 2014).

One alternative/complement to antibiotic therapy is the use of bacteriophages. Bacteriophage (or phage) therapy utilizes naturally occurring lytic bacteriophages (viruses that infect and lyse bacteria), which can be isolated from common environmental reservoirs, to target and destroy pathogenic bacteria in a human host. Though numerous promising reports of phage therapy have been published, they have, to date, not resulted in common use in the United States or much of the West currently (Carlton, 1999; Sulakvelidze and Pasternack, 2009; Fair and Tor, 2014; Lin et al., 2017). Structurally, lytic bacteriophages are comprised of DNA encapsulated by a protein capsid, rendering them non-toxic with regard to human use (Loc-Carrillo and Abedon, 2011). Moreover, bacteriophages are highly specific, infecting only a narrow range of targeted bacterial strains, generally resulting in minimal distortion of the surrounding microbiome community (Matsuzaki et al., 2005; Skurnik et al., 2007; Kutter et al., 2010; Mai et al., 2010, 2015; Cieplak et al., 2018). Bacteriophages are found in prodigious numbers in the environment. They have been isolated from marine environments (both saltwater and freshwater), various food sources (including fish, dairy products, and fruits and vegetables), soils, plants, and other environmental sources (Sulakvelidze et al., 2001; Kutateladze and Adamia, 2010). Phages are also common commensals of human body, and can be commonly isolated from human skin, vagina, mouth and rest of the gastrointestinal tract where their numbers are estimated to be ca. 1 × 10^15^ (Sulakvelidze and Kutter, 2005; Dalmasso et al., 2014). As new sources for novel antibiotics have become limited, the advantage of (i) bacteriophages being the most abundant organisms within the planet’s biosphere, and (ii) them having a different (compared to antibiotics) mechanism of action for killing bacteria, should be further explored (Alisky et al., 1998). Interestingly, several bacteriophage products targeting *Listeria monocytogenes*, *Salmonella* spp., *Shigella* spp., and *Escherichia coli* (*E. coli*) O157:H7 recently received Generally Recognized as Safe (GRAS) status from the FDA, and they are increasingly utilized by the food industry in the United States for improving the safety of various foods, including ready-to-eat foods (Clokie et al., 2011; Chang et al., 2014; Moye et al., 2018).

The human gastrointestinal (GI) tract is diversely colonized by abundant symbiotic microbiota, playing critical roles in immunomodulation, digestion of various compounds, and protecting against negative health outcomes (Thursby and Juge, 2017). Most organisms colonizing the human body are beneficial, however, some can transition out of a commensal relationship into a pathogenic one under particular circumstances, though it is not fully understood why (Avila et al., 2009). Importantly, these native pathobionts are not without their respective complements, as the body also hosts numerous bacteriophages, though the influence these natural phages have on human health requires further investigation (Navarro and Muniesa, 2017). Research has recognized the relationship between health and the gut microbiome in various infections and non-infectious diseases, including inflammatory bowel diseases, obesity, and various metabolic disorders (Sheflin et al., 2014; Jandhyala et al., 2015). As the GI tract is inhabited by an estimated ∼100 trillion commensal bacteria, maintenance of the microbial balance is complex (Haque and Haque, 2017), and it can be distorted by many factors including shifts in diet, stress, or environmental changes (Antonopoulos et al., 2009; Sweeney and Morton, 2013). A noteworthy source of profound gut microbiome dysbiosis can be exposure to antibiotics (Theriot et al., 2014). Due to the broad spectrum “wipe-out” approach of antibiotic therapy, unintended beneficial microorganisms are also targeted alongside intended enteric pathogens (Nelson and Levy, 2011; Chandel and Budinger, 2013). This non-specific expulsion of microbiota (“collateral damage”) creates voids in the gastrointestinal tract, which leave the host vulnerable to colonization or infection of new harmful microorganisms which were previously prevented by symbiotic gut bacteria (Theriot et al., 2014; Coryell et al., 2018). Thus, antimicrobial approaches that enable targeted, specific elimination of problem bacteria while preserving the normal microflora of the GI tract may be invaluable. Bacteriophages may provide one such approach, and it could be especially effective for managing foodborne bacterial infections.

Foodborne infections continue to be a major public health concern (Switaj et al., 2015). Among a handful of bacterial foodborne pathogens, Shiga toxin-producing *E. coli* (STEC or *enterohemorrhagic E. coli*) – and especially *E. coli* O157:H7 – are among the most common and deadly bacterial pathogens associated with foodborne outbreaks in North America (Centers for Disease Control and Prevention, 2018). These bacteria can cause diarrhea, hemorrhagic colitis, hemolytic uremic syndrome (HUS), and thrombotic thrombocytopenic purpura, with the latter two complications having high risks of fatality (Rahal et al., 2015). Antibiotics have been shown to aggravate Shiga toxin production, increasing the risk of developing severe HUS or TTP (Bielaszewska et al., 2012) resulting in symptom management and hydration therapy being the primary courses of action. Patients can be in extended discomfort until the infection resolves, emphasizing the need for effective pathogen-reducing agents as alternatives to antibiotics. As noted above, bacteriophages may provide one such alternative/complementing prevention and/or treatment modality which could be used either preventively or therapeutically (e.g., to enhance the normal human gut microbiota with STEC-specific bacteriophages, to kill any enterohemorrhagic *E. coli* that may be introduced into human GI tract with contaminated food, before they cause disease). The purpose of this study was to (i) investigate the efficacy of an *E. coli* O157:H7*/Salmonella* spp*./L. monocytogenes*-targeting bacteriophage cocktail (tentatively named F.O.P.) in reducing the levels of a specific enterohemorrhagic *E. coli* strain (*E. coli* O157:H7) in experimentally infected mice, and (ii) determine how treatment with F.O.P. impacted the normal gut microbiota when compared with a commonly used for managing *E. coli* infections antibiotic ampicillin.

## Materials and Methods

### Bacteriophage Formulation

Three types of bacteriophage treatments were included in this study: (1) the multi-target bacteriophage preparation tentatively named “Foodborne Outbreak Pill” (F.O.P.) – a combination of *E. coli* O157:H7, *Salmonella* spp., and *L. monocytogenes*-targeting lytic bacteriophages, (2) the F.O.P. bacteriophage preparation diluted by a factor of 10 with standard phosphate buffer solution (PBS), labeled F.O.P. 1:10, and (3) an *E. coli* O157:H7-specific bacteriophage preparation labeled as EcoShield PX^TM^ (the *E. coli* phage component included in F.O.P.). All bacteriophage formulations were provided by Intralytix, Inc.

The F.O.P. bacteriophage cocktail has been described previously (Moye et al., 2019). Briefly, F.O.P. is a combination of three FDA- and/or USDA-evaluated, commercial phage preparations for food safety applications in the United States, Canada, and Israel: ListShield^TM^ (six lytic phages), EcoShield PX^TM^ (three lytic phages) and SalmoFresh^TM^ (six lytic phages). The current F.O.P. phage preparation components are summarized in Table 1. Each of the bacteriophage preparations constituting the F.O.P. preparation was diluted from a high-titer stock to obtain a concentration of approximately 1 × 10^10^ PFU/mL prior to mixing. Mice were treated with 0.1 mL, thus achieving a therapeutic dose of 1 × 10^9^ PFU. The bacteriophage preparations were stored at 2–8°C in dark until used.

**TABLE 1:** Bacteriophage preparations used to create the multi-target F.O.P. bacteriophage preparation.

**SalmoFresh^TM^**	**ListShield^TM^**	**EcoShield PX^TM^**
*Salmonella* phage SBA-1781	*Listeria monocytogenes* phage LMSP-25	*Escherichia coli* O157:H7 phage ECML-359
*Salmonella* phage SKML-39	*Listeria monocytogenes* phage LMTA-34	*Escherichia coli* O157:H7 phage ECML-363
*Salmonella* phage SPT-1	*Listeria monocytogenes* phage LMTA-148	*Escherichia coli* O157:H7 phage ECML-117
*Salmonella* phage SSE-121	*Listeria monocytogenes* phage LMTA-57	
*Salmonella* phage STML-13-1	*Listeria monocytogenes* phage LMTA-94	
*Salmonella* phage STML-198	*Listeria monocytogenes* phage LIST-36	

### Challenge *E. coli* Strain

We used *E. coli* O157:H7 nalidixic acid resistant (NalAc^R^) strain Ec231 as the target pathogen (Carter et al., 2012). Our challenge *E. coli* strain is pathogenic in humans but not in mice, and it is sensitive to the EcoShield PX^TM^ bacteriophage preparation, which is also included in the multi-target F.O.P. bacteriophage preparation. We diluted early log phase *E. coli* O157:H7 in LB broth to challenge with 1 × 10^8^ *E. coli* O157:H7 CFU per mouse.

### Mouse Study

**FIGURE 1:**
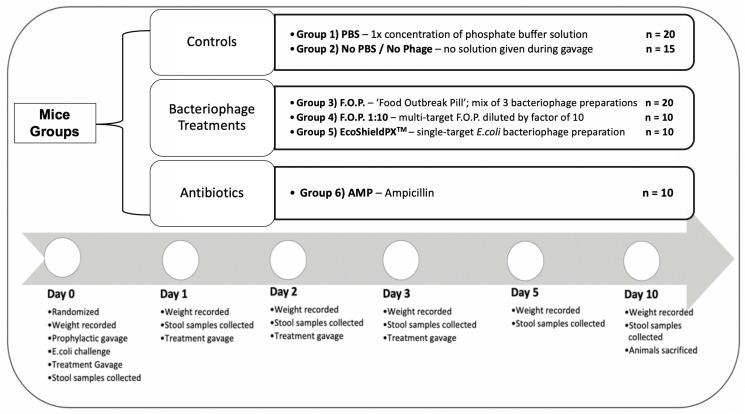
Top of figure denotes randomized mice groups and category of treatment each group belongs in. Bottom of figure denotes timeline of observation from the start of each experiment on Day 0, to the end of each experiment on Day 10.

For the duration of the study a total of 85 C57BL male mice (8 weeks old) acquired from The Jackson Laboratory (JAX) were acclimated for 7 days in the University of Florida’s (UF) Animal Care Services (ACS) facility. They were randomized by cage into six groups for 10 days of observation. Each cage contained five animals and each group was assigned to either a specific treatment therapy or control group to combat the challenge of *E. coli* O157:H7 bacteria. A total of six experiments were conducted consecutively, with no more than three cages per experiment, to reach the total sample size (Figure 1).

Mice were kept under SPF (specific pathogen free) conditions, thus, controls would not harbor the challenge *E. coli* O157:H7 strain. The initial challenge and all subsequent therapies were administered via oral gavage to achieve standardized dosing. Oral gavage was performed using appropriately sized sterilized gavage needles. Animals were sacrificed on the final experiment day in accordance with standard protocols provided by the University of Florida’s Institutional Animal Care and Use Committee (IACUC) and ACS guidelines. Each experiment followed a standardized timeline summarized in Figure 1.

#### Treatment Groups

Group 1 was a control group that received neither phosphate buffer solution (PBS, pH 7.4) nor any bacteriophage therapy but received the oral gavage procedure to standardize stress levels and was labeled No PBS/No Phage. Group 2 was a control group that received only PBS by oral gavage. Group 3 received normal strength F.O.P. therapy. Group 4 received F.O.P. 1:10 therapy. Group 5 received normal strength EcoShield PX^TM^. Group 6 received ampicillin (AMP).

On Day 0, mice were individually weighed, and ear punched for identification, followed by three oral gavage procedures. All gavage procedures were separated by a 2.5 h waiting period between each procedure. Mice received a prophylactic gavage, the *E. coli* challenge gavage, and a treatment gavage. The prophylactic gavage was the therapy assigned at random to each cage. Mice were then challenged with 0.2 mL fresh 4-h culture of *E. coli* O157:H7 nalidixic acid resistant (NalAc^R^) strain Ec231 to achieve a dose of ca. 1 × 10^8^ CFU *E. coli* in each animal. 2.5 h post-challenge immediately prior to administering respective therapy, stool samples were collected from all mice. All treatments, excluding ampicillin, were administered twice daily by oral gavage for four consecutive days (Days 0, 1, 2, and 3). Ampicillin was administered twice on Day 0 (before and after bacterial challenge) and not on any other day. After Day 0, mice in the ampicillin group received gavage procedures without solution to standardize stress.

#### Sample Collection

Stool samples were collected on Days 0, 1, 2, 3, 5, and 10. Each sample contained three fecal pellets of approximately 50 mg that were homogenized by glass beads in 0.45 ml of PBS.

### Plate Counts for *E. col*i

To selectively support growth of the challenge *E. coli* O157:H7 strain while reducing growth of other microorganisms, freshly prepared LB Agar plates containing nalidixic acid (NalAc at 25 μg/ml) were used.

We diluted 0.1 ml of each sample by a factor of 10 and a factor of 100. We plated the dilution on LB Agar + NalAc plates in duplicates and after a standard incubation period of 24 h at 37°C, viable *E. coli* O157:H7 counts were recorded by sample collection date for each animal. Limited fecal suspensions were taken during plating to preserve enough original sample for subsequent laboratory analysis. The weight of each animal was recorded prior to initial challenge, at every stool collection, and prior to sacrificing for trend analysis.

### *E. coli* Genome Copy Analysis

For DNA extraction, 0.1 ml suspensions from individual fecal samples were aliquoted into a combined cage sample (total volume 0.5 ml) using a cut pipette tip. DNA was extracted using a modified Qiagen stool DNA protocol (Mai et al., 2006). DNA was amplified by qPCR using sequencing primers (1Slt224 gene *stx1* ATG TCA GAG GGA TAG ATC CA) and (1Slt385 gene stx1TAT AGC TAC TGT CAC CAG ACA AT) to determine the number of *E. coli* O157:H7 genome copies within treatment groups for Days 0, 1, 2, and 3.

### Denature Gradient Gel Electrophoresis Microbiota Profiling

We initially analyzed microbiota community profiles using DGGE (Denature Gradient Gel Electrophoresis). A 457-bp fragment from the V6 to V8 region of the bacterial 16S rDNA gene was amplified with primers U968-GC and L1401. DGGE was performed in an 8% (wt/vol) polyacrylamide gel with a denaturing gradient ranging from 40 to 50% at the top and bottom of the gel, respectively (100% denaturing conditions were defined as 7 M urea and 40% formamide). After electrophoresis (16 h, 65 V, 60°C), the gel was stained with SYBER Green (Novex, San Diego, CA, United States). DGGE analysis was performed only for F.O.P. bacteriophage therapy, ampicillin therapy, and PBS therapy groups for Days 0, 1, 5, and 10. We compared the degree of microbiota distortion between treatment groups by comparing banding patterns indicative of microbiota diversity within each group and evaluating levels of band conservation. Distortion for both F.O.P. therapy and ampicillin therapy were compared against the PBS control group. We did not evaluate microbiome profiles for the EcoShield PX^TM^ bacteriophage therapy or the diluted F.O.P. 1:10 bacteriophage therapy.

### 16S DNA Library Preparation and Sequencing

Samples of mouse fecal homogenates were delivered to Genewiz, Inc (Suzhou, China) where the 16S libraries were constructed and sequenced. The sequencing and bioinformatics workflows were designed and carried out by Genewiz, Inc., Briefly, genomic DNA was extracted from the homogenates using the DNeasy PowerSoil Kit (Qiagen) according to the manufacturer’s instructions and measured using a Qubit 2.0 Fluorometer (Invitrogen, Carlsbad, CA, United States). 30–50 ng of extracted DNA was used to prepare amplicons via the MetaVx^TM^ library preparation kit (Genewiz, Inc., South Plainfield, NJ, United States) following the manufacturer’s instructions. 16S sequencing was performed on the V3 to V4 region of the 16S ribosomal DNA. Primers were optimized for use by Genewiz, Inc., and the sequence of the forward and reverse primers are CCTACGGRRBGCASCAGKVRVGAAT and GGACTACNVGGGTWTCTAATCC, respectively. After an initial round of PCR, a second round of amplicon sequencing was carried out to enrich the samples. During this second round of sequencing, indexing adaptors were added to the amplicons to prepare the libraries for next generation sequencing. The quality of the DNA libraries was assessed using an Agilent 2100 Bioanalyzer (Agilent Technologies, Palo Alto, CA, United States), and the quantity of DNA was determined using a Qubit 2.0 Fluorometer. Next generation sequencing was performed on multiplexed DNA libraries using an Illumina MiSeq (Illumina, San Diego, CA, United States), which was operated following the manufacturer’s guidelines. A total of 2 × 250 paired-end read cycles were performed. The MiSeq Control Software within the sequencing instrument was used for image analysis and base calling.

### Bioinformatics

Analysis of the 16S rRNA metagenomic data was performed using the QIIME bioinformatics pipeline (Caporaso et al., 2010). Pair-end reads were joined and trimmed to remove the barcodes and primer sequences, and the joined reads were grouped into samples based on these barcodes. Reads that were shorter than 200 bp, that contained ambiguous bases, or with a mean quality score below 20 were removed from further analysis. Chimeric sequences were detected and removed using a reference database (RDP Gold) employing the UCHIME algorithm. Following these quality control steps, each sample contained an average of 71,751.5 counts (max: 97,134; min: 40,487). The sequenced reads for each sample were then subsampled with a maximum value for sampling set to the sample containing the least number of counts (40,487), and the clustering program VSEARCH (version 1.9.6), set to apply the Silva 119 database pre-clustered at 97% sequencing identity, was used to assign sequenced reads to Operational Taxonomic Units (OTUs). A confidence threshold of 0.8 was set for the Ribosomal Database Program (RDP) classifier, and the taxonomical predictions derived from the Silva 119 database were assigned at the species level. Once the binning was completed, family (and genus)-level heatmaps based on the relative abundance were prepared from this data using the QIIME pipeline, and modified genus- and family-level heatmaps were generated using the relative abundance datasets.

Prior to alpha and beta diversity calculations, the dataset underwent a second round of ratification. The QIIME pipeline was used to calculate ACE, Chao1, Shannon, and Simpson estimators of alpha diversity (Figure 8). A Principle Coordinate Analysis (PCoA) of the beta diversity was also prepared using the QIIME bioinformatics pipeline (Figure 9).

### Statistical Analysis

To analyze viable count data, we performed two-tailed *t*-tests to determine statistical significance for both separate experiments and across all groups using data from all the experiments combined together. A two-way ANOVA with Tukey’s *post hoc* test was conducted using GraphPad Prism to determine the significance of changes in the alpha diversity of the bacterial microbiomes from mouse fecal homogenates. Weight data was evaluated by determining percent change of individual mice. Weight measurements taken prior to the start of each experiment (on Day 0 after 7 days of acclimation) were used as baseline for assessment.

## Results

### Effects of Bacteriophage on Fecal *E. coli* Recovery

**FIGURE 2:**
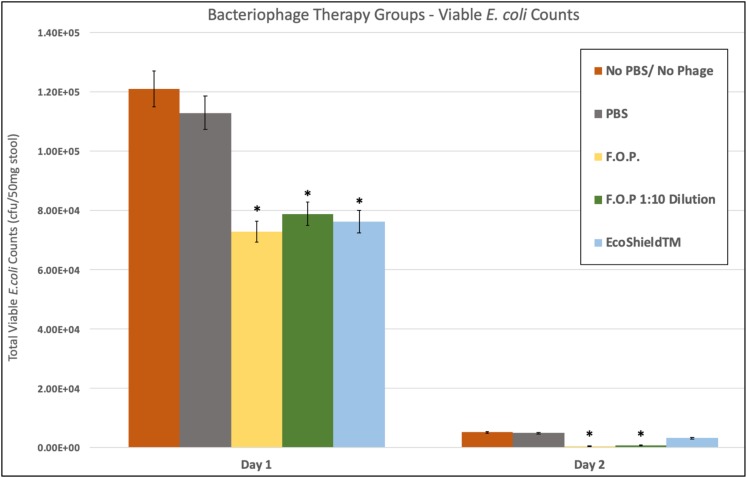
Comparison of different phage therapies. Day 1 and Day 2 results for control groups and bacteriophage treatment groups. Denotes the combined pathogen reduction data of separately conducted Experiments 1, 2, 3, and 4. Absolute numbers are used on the *y*-axis scale. Bars marked with ^∗^ indicate statistically significance when compared against the PBS control group.

**FIGURE 3:**
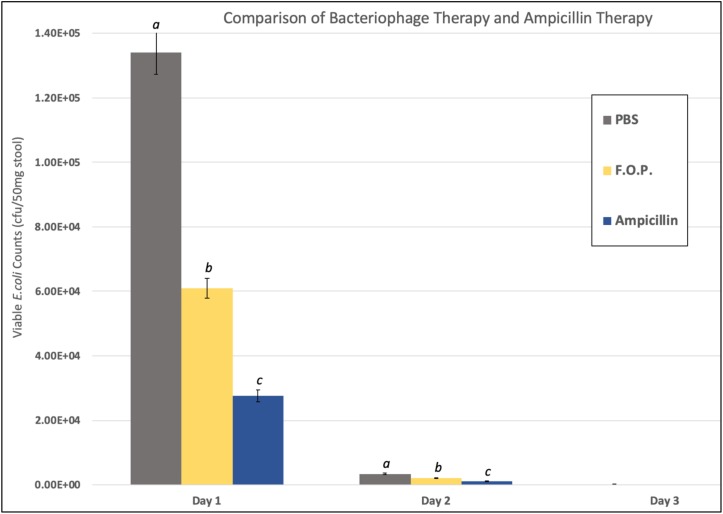
Comparison of F.O.P. bacteriophage therapy with ampicillin therapy. Denotes Day 1, Day 2, and Day 3 data combined for all experiments evaluating PBS, F.O.P., and Ampicillin together. *Y*-axis uses a log scale of viable *E. coli* counts for the best visualization of the data trend. Designations of a, b, and c indicate separate statistical significance of viable *E. coli* counts for F.O.P. and Ampicillin against the control group PBS.

Viable *E. coli* O157:H7 counts were reduced in all three bacteriophage treatment groups on Days 1 and 2 post-challenge, with an observed dose response for F.O.P. (Figure 2). By Day 3 viable *E. coli* O157:H7 counts dropped below the detection thresholds for all groups that received bacteriophage therapy. In separate experiments the F.O.P. bacteriophage reduced viable counts by 54% and the antibiotic treatment (ampicillin) reduced viable counts by 79% with both significant reductions in *E. coli* O157:H7 counts occurring by Day 1 (Figure 3).

**FIGURE 4:**
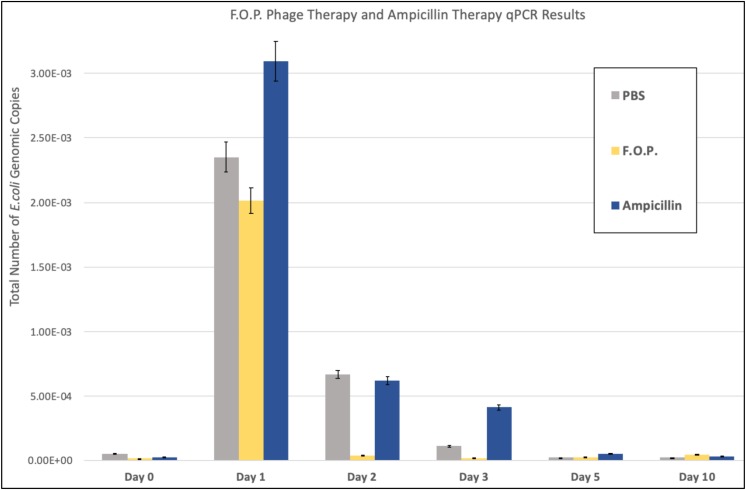
qPCR results. The total number of *E. coli* genomic copies is along the *y*-axis. Denotes the number of *E. coli* genomic copies within F.O.P., Ampicillin, and PBS, graphed on the *x*-axis according to experiment day.

Quantification of fecal *E. coli* Ec231 by qPCR, which targets DNA from both viable as well as dead *E. coli*, confirmed the trends observed for viable plate counts (Figure 4). qPCR was performed on samples belonging to PBS, F.O.P. and ampicillin groups only.

### Effects of Treatment With Phages or Ampicillin on Gut Microbiota

**FIGURE 5:**
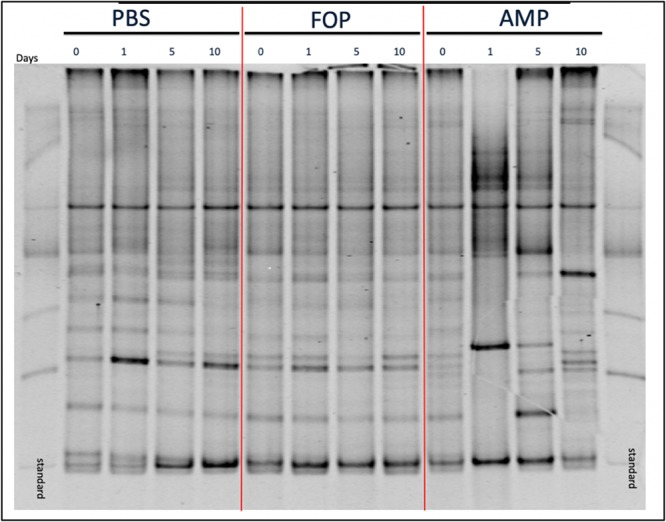
DGGE Image of Microbiota Profile. DGGE Analysis of combined cage samples denoting the gut microbiome profiles between PBS, F.O.P. phage therapy, and ampicillin therapy groups during the experiments over the course of 10 days.

To determine changes in microbiota associated with various treatments we performed (i) Denaturing Gradient Gel Electrophoresis (DGGE), followed by (ii) 16S rRNA sequencing. DGGE revealed a large change in the microbiota for the ampicillin group that only partially recovered 10 days post-treatment. In contrast, DGGE showed consistent microbiota profiles (i.e., no significant changes) for both the PBS and the F.O.P. therapy groups (Figure 5). We further examined, through 16S rRNA sequencing, the bacterial microbiota of fecal homogenates of the mice (using four mice from each group) for Days 0, 1, 5, and 10.

**FIGURE 6:**
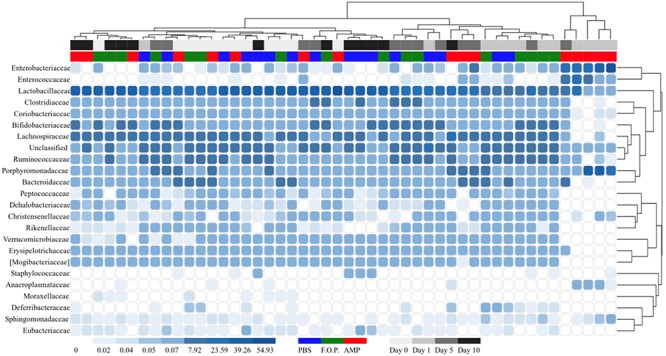
Family level heatmap (sorted by hierarchy). Displays the taxa identified in mouse fecal homogenates by hierarchy. Heatmaps were generated representing the relative abundance of taxa assigned to OTUs at the family level. The columns were sorted by the treatment type, individual mouse, and day post-treatment.

**FIGURE 7:**
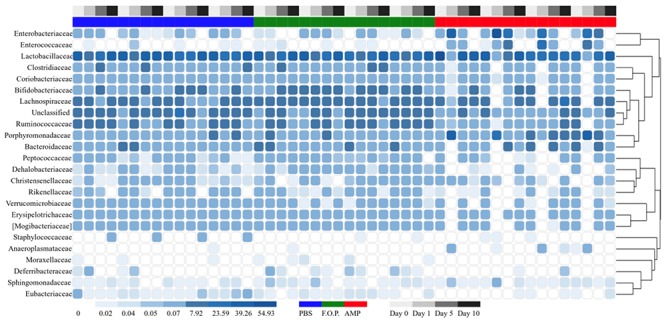
Family level heatmap (sorted by time). Displays the taxa identified in mouse fecal homogenates in chronological order of experiment days. Heatmaps were generated representing the relative abundance of taxa assigned to OTUs at the family level. The columns were sorted by the treatment type, individual mouse, and day post-treatment.

For our microbiome analysis of mouse fecal homogenates, we first examined the relative abundance of bacterial taxa at the genus level using heat maps organized by hierarchy (Supplementary Figure A1) and time (Supplementary Figure A2). We observed that microbiomes of mice treated with ampicillin changed drastically within 1 day after receiving the ampicillin. The bacterial microbiota 1 day after ampicillin treatment (and in some cases 5 days) formed a distinct branch apart from the remaining samples in our study (Supplementary Figure A1). While this genus-level approach reveals finer detail regarding shifts in bacterial organisms, we still observed a high level of unclassified taxa for samples. For this reason, we also examined family-level heatmaps of bacterial microbiomes from mouse fecal homogenates. Similar to the genus-level data, we observed a clustering of samples from the ampicillin group on Day 1 indicating a collapse in the microbiota (Figures 6, 7). Compared to both PBS- and phage-treated mice, there was greater distortion of microbiota in the ampicillin group throughout the study period. A consistent effect of ampicillin treatment was an enrichment in *Enterobacteriaceae*, *Enterococcaceae*, and *Porphyromonadaceae* on Day 1 and Day 5 when compared to other treatment groups (Figure 6). During the course of the experiment, the microbiota of the mice treated with ampicillin were gradually restored, but this process was individual-specific with some mice still displaying clear dysbiosis 5 days post-treatment (Figure 6).

**FIGURE 8:**
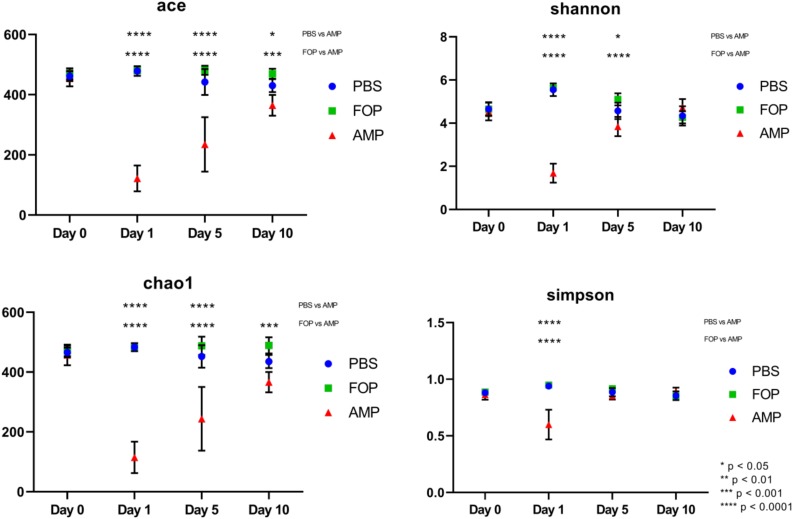
Alpha Diversity (ACE, Chao1, Shannon, and Simpson Diversity Index). Alpha diversity measures of mouse fecal homogenates at day 0 and 1, 5, and 10 days post-treatment. The alpha diversity of the communities present in mouse fecal homogenates were assessed using the ACE, Chao1, Shannon, and Simpson diversity estimators. Data are the mean of four individuals, and error bars represent the standard deviation. Significant changes, as determined by a two-way ANOVA with Tukey’s *post hoc* test, are signified using asterisks. No significant changes were detected between FOP and PBS-treated mice.

A striking change in the relative abundance of microbial taxa of ampicillin treated groups was a general scarcity of all but a few members of the initial bacterial microbiome. To quantitatively examine the effect of each treatment on the diversity of the mouse gut microbiota, we calculated the alpha diversity using four common diversity estimators. This was done to avoid bias present in any single measure. By each estimator of diversity, we observed a significant drop in alpha diversity 1 day after treatment with ampicillin (Figure 8), with a gradual partial return to the levels of diversity present in the other treatment groups. This was not the case in phage-treated samples where the alpha diversity by each of the measures virtually mirrored the diversity we observed for the PBS-treated mice. Importantly, no significant difference between the PBS and F.O.P.-treated samples was observed by any of the estimators of community richness used.

**FIGURE 9:**
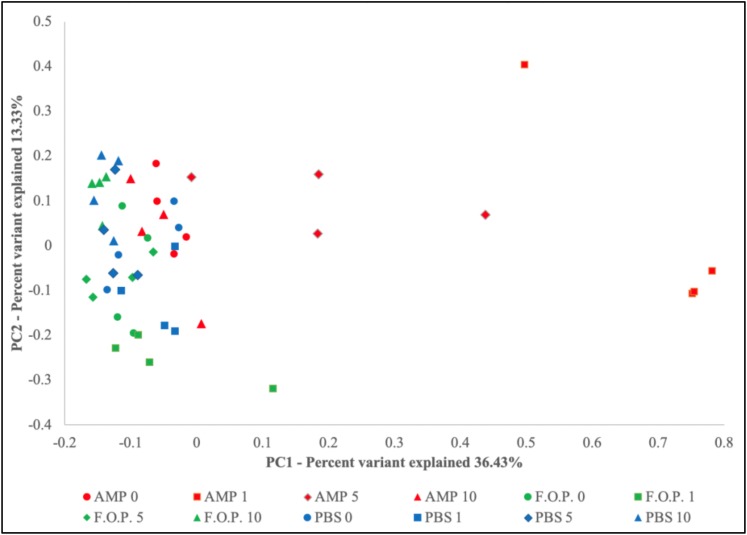
Principle Coordinate Analysis (PCoA) Graph of the Beta Diversity. The beta diversity of communities present in the mouse fecal homogenates was visualized using a principle coordinate analysis plot comparing PC1 (explaining 36.43%) and PC2 (explaining 13.33%).

Based on the hierarchical sorting of microbial taxa in our family and genus-level heatmaps (Figure 6 and Supplementary Figure A1), it appeared that the major change between the bacterial microbiome of the mouse fecal homogenates occurred primarily on Day 1 in groups treated with ampicillin, with a few cases extending through Day 5. To examine the relatedness of these samples in closer detail, we plotted the beta diversity by PCoA, and the variable that most easily explains the first percent variant (explaining 36.43% of the diversity) were samples from mice 1 day after ampicillin treatment and, to a lesser extent, a few of the mice 5 days after treatment with ampicillin (Figure 9), supporting our initial observation (Figure 6). In contrast, the samples treated with PBS and F.O.P. remained closely associated across all experiment days. No other clear patterns in the relatedness of samples belonging to any treatment groups other than ampicillin were immediately apparent.

### Weight Change Observations

**FIGURE 10:**
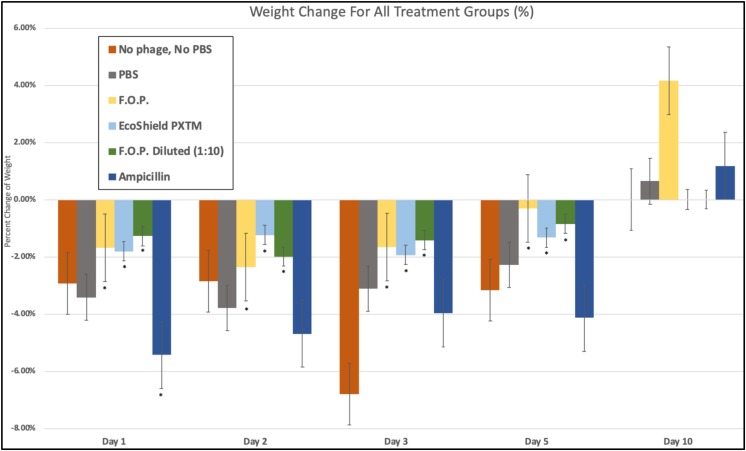
Weight fluctuations across all therapy groups. Weight changes were calculated as percent change from baseline weight on Day 0 through the 10 days of each experiment. The bacteriophage treatment groups experienced the least amount of weight loss, where ampicillin experienced the greatest initial weight loss. Bars marked ^∗^ indicate statistical significance when compared against PBS control groups.

We observed weight loss in all groups on Day 1, 24 h after the initial challenge on Day 0. Over this 24-h period, the bacteriophage groups had minimal average weight loss (calculated as percent change from Day 0 weight) when compared to control and antibiotic groups (*p* < 0.05) (Figure 10).

Day 1 weight changes observed in all bacteriophage and antibiotic treated groups were statistically significant against the PBS control groups. The ampicillin group had the greatest weight loss with a 5.44% reduction for Day 1 and continued to have the greatest weight loss of all groups for Days 2, 3, and 5. Within the bacteriophage therapy groups, EcoShield PX^TM^ had the greatest weight loss observed for Day 1, with F.O.P. therapy having the greatest weight loss on Day 2. The weight loss values of F.O.P. 1:10 stayed close to, but lower than, the F.O.P. therapy group on all days except for Day 5 where F.O.P. 1:10 groups had slightly more weight loss. Weight loss was observed for all groups through Day 5. By Day 10 mice displayed some recovery of weight loss, with F.O.P. showing the greatest recovery with an average weight gain of 4.15% compared to Day 0 baseline weight (Figure 10).

## Discussion

Consistent with previous observations (Smith and Huggins, 1982; Barrow et al., 1998; Chibani-Chennoufi et al., 2004), all bacteriophage formulations reduced viable counts of the targeted *E. coli* O157:H7 strain. The reductions for different therapy groups were similar from Day 3 onward. However, counts from Day 1 and 2 indicate that the F.O.P. therapy was more effective than EcoShield PX^TM^ or 1:10 diluted F.O.P. therapies. F.O.P. resulted in fewer viable *E. coli* counts over the course of Days 1, 2, and 3. Although reductions in viable *E. coli* counts in the ampicillin-treated mice were numerically slightly better compared to F.O.P.-treated mice, the differences were not statistically significant – suggesting that the efficacy of F.O.P. against the challenge *E. coli* strain was essentially the same as that of ampicillin.

Based on the results of viable *E. coli* counts between F.O.P. and F.O.P. 1:10, there appeared to be a dose-effect between pathogen reduction and concentration of the F.O.P. bacteriophage therapy (with higher F.O.P. concentrations being more effective). There is an observable trend of lower viable *E. coli* O157:H7 counts in F.O.P. on Day 1, 2, and 3, compared to the counts of 1:10 diluted F.O.P. though the count difference between these two phage therapy groups did not have statistical significance. The observation suggest that phage treatment is concentration-dependent but allows some flexibility in phage titers for sustainable efficacy. Additional dose deescalating studies will be required to better delineate the minimal effective dose.

Assessing trends in weight data for both F.O.P. and 1:10 diluted F.O.P. revealed that the F.O.P. therapy groups did have greater weight loss than the 1:10 diluted F.O.P. groups for Days 1, 2, and 3, supporting the viable count reduction trend we observed. Both the multi-target F.O.P. bacteriophage preparation and the single target EcoShield PX^TM^ reduced the *E. coli* counts with similar efficacy for Day 1; however, on Day 2 the F.O.P. showed stronger efficacy. We assessed for signs of batch effect between different experiment cycles, experiment Days, and animal cages through two-tailed *t*-tests. Based on analysis for data testing both F.O.P. and EcoShield PX^TM^ in the same cycle, we found statistical significance in the viable count reduction difference on Day 2, suggesting the multi-target F.O.P. had greater efficacy against the single-target EcoShield PX^TM^ on that day. The reasons behind this phenomenon are unclear but could be due to some unintended cross-kill of the targeted *E. coli* O157:H7 population by the *Salmonella* phages in F.O.P., or some other synergistic effect. It is also possible that this was due to fluctuation in *E. coli* counts that happened to reach significant levels on that day of treatment. Additional studies with larger number of animals and de-escalating doses of treatments will be required to further elucidate this observation.

The number of *E. coli* genomic copies (determined through qPCR) were greater in the ampicillin therapy group when compared to F.O.P. and control groups. This might be due to ampicillin inhibiting but not as efficiently lysing the challenge strain compared to bacteriophage. Lysis of the target *E. coli* O157:H7 leads to DNA degradation by DNAses present in the gut and less template available for PCR.

The results of our microbiota analysis demonstrated that, while F.O.P. and ampicillin were similarly effective at reducing the levels of their targeted *E. coli* O157:H7 strain in mice, the F.O.P. bacteriophage preparation was noticeably better in maintaining the natural richness and diversity of the gut commensal flora compared to ampicillin. These results are consistent with previous investigations wherein a *Listeria*-specific and *Shigella*-specific bacteriophage cocktail were shown in separate instances to be as effective as a broad-spectrum antibiotic at reducing the fecal counts of *L. monocytogenes* and *Shigella sonnei* in infected mice (Mai et al., 2010, 2015). Also, in another study with an *in vitro* human colon simulator model system, an *E. coli*-specific phage preparation performed similarly, and in some cases better than, the antibiotic ciprofloxacin at reducing the counts of *E. coli* after a simulated infection (Cieplak et al., 2018). In agreement with our results presented here, antibiotic treatment in that previous study (Cieplak et al., 2018) also suppressed the defined bacterial consortia of the simulated gut, while this detrimental effect was not observed after phage treatment (Cieplak et al., 2018). As with any therapy, there is understandable concern for future development of resistance. The introduction of non-native phages to a body comes with the possibility of forming phage-resistant bacterial mutants, however, the practical likelihood of this resulting in a negative health impact is yet to be determined. Available literature suggests bacterial mutants are unlikely to provide robust protection against therapeutic phage cocktails, as the antagonistic coevolution between bacterial strains and respective phage strains is a driving force of microbial diversity, and phages adapt as rapidly as mutations occur (Sulakvelidze and Kutter, 2005). Taken together, these studies comparing the effectiveness of broad-spectrum antibiotics and pathogen-targeting phage preparations demonstrate that both treatments are approximately equally effective at reducing the levels of their targeted bacteria, but phage treatments provide much milder (essentially undetectable) impact on the overall commensal – and often beneficial – bacterial microbiome compared to antibiotic treatments. These data provide further support to the idea that carefully designed cocktails of lytic bacteriophages could be useful for gently fine-tuning the gut microbiota for preventing or treating foodborne infections, by specifically reducing or eliminating targeted bacterial pathogens in the gastrointestinal tract without deleteriously impacting the normal gut microflora.

## Conclusion

We observed three main effects of the multi-target bacteriophage cocktail (F.O.P.): (1) F.O.P. was effective in significantly reducing the levels of an enterohemorrhagic *E. coli* O157:H7 strain in infected mice; the effectiveness was approximately the same at that observed with ampicillin (statistically not significantly different); (2) F.O.P. had much milder (essentially undetectable) impact on the normal gut microbiota composition compared to ampicillin which showed significant disruption of gut microbiota as early as the first day of treatment; and (3) The F.O.P. bacteriophage treatment had no deleterious impact on the weight of mice (the smallest percent of weight change); in contrast, the greatest initial weight loss was observed was the antibiotic treatment group. In summary, prophylactic or therapeutic applications of the F.O.P. or similar lytic phage bacteriophage preparations may be useful for preventing or treating gastrointestinal bacterial infections – including those caused by consuming foods contaminated with major foodborne bacterial pathogens such as *L. monocytogenes*, *Salmonella* spp. and enterohemorrhagic *E. coli* (e.g., *E. coli* O157:H7) – without any deleterious impact on the normal – and often beneficial – gut microflora (Sulakvelidze and Kutter, 2005). Bacteriophages can also provide an invaluable supplemental/complementing tool for managing drug-resistant bacterial infections and/or for gently fine-tuning the mammalian microbiome (by specifically targeting problem bacteria without deleteriously impacting the commensal microflora) in order to provide various health benefits (Mai et al., 2010, 2015; de Gunzburg et al., 2017).

## Data Availability

Metagenomic data generated for this study is available in the National Center for Biotechnology Information (NCBI) repository under BioProject PRJNA560178.

## Ethics Statement

This study used 85 C57BL male mice (8 weeks old) acquired from The Jackson Laboratory (JAX) were acclimated for 7 days in the University of Florida’s (UF) Animal Care Services (ACS) facility. This study (#201810231) was approved by the University of Florida’s Institutional Animal Care and Use Committee (IACUC) in 2018.

## Author Contributions

All authors listed have made a substantial, direct and intellectual contribution to the work, and approved it for publication.

## Conflict of Interest Statement

AS owns equity in the company Intralytix, Inc. ZM was an employee of the company Intralytix, Inc. at the time of the study.

The remaining authors declare that the research was conducted in the absence of any commercial or financial relationships that could be construed as a potential conflict of interest.
