# Supplementary material for: Bacteriophages Reduce Pathogenic Escherichia coli Counts in Mice Without Distorting Gut Microbiota
Source: Front Microbiol. 2019 Sep 10;10:1984. doi: 10.3389/fmicb.2019.01984 (PMC6748168; doi:10.3389/fmicb.2019.01984)
Supplement: Supplementary file 1 [file Data_Sheet_1.docx]

Supplementary Material

# Supplementary Figures

From the same 16S rRNA sequencing data used to generate the family level heatmaps found in the main text of the manuscript, we also generated two genus level heatmaps as supporting data for the findings of this study.

## Supplementary Figure A1


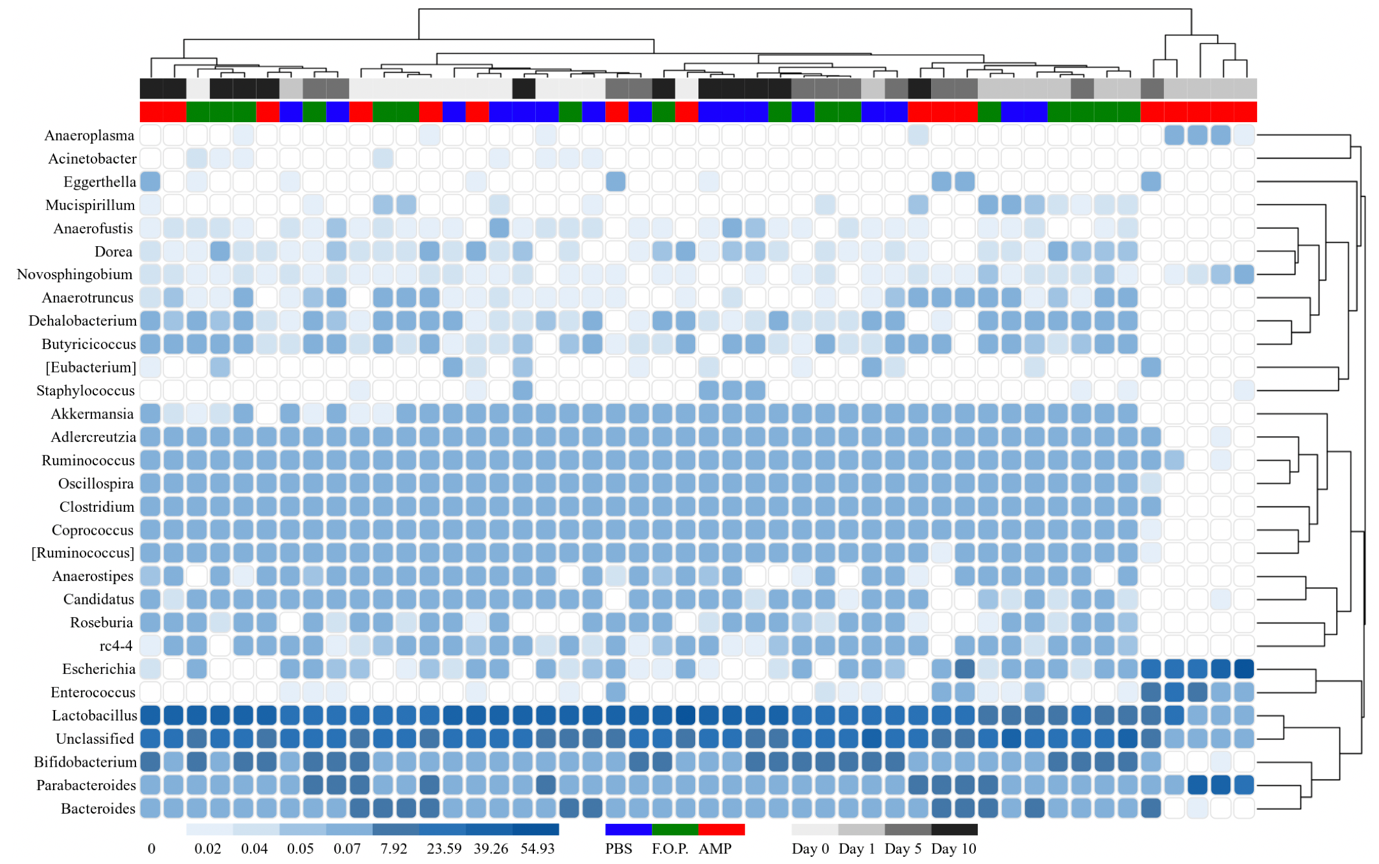


**Supplementary Figure A1.** Genus level heatmap (sorted by hierarchy). Displays the taxa identified in mouse fecal homogenates by hierarchy. Heatmaps were generated representing the relative abundance of taxa assigned to OTUs at the genus level. The columns were sorted by the treatment type, individual mouse, and day post-treatment.

## Supplementary Figure A2


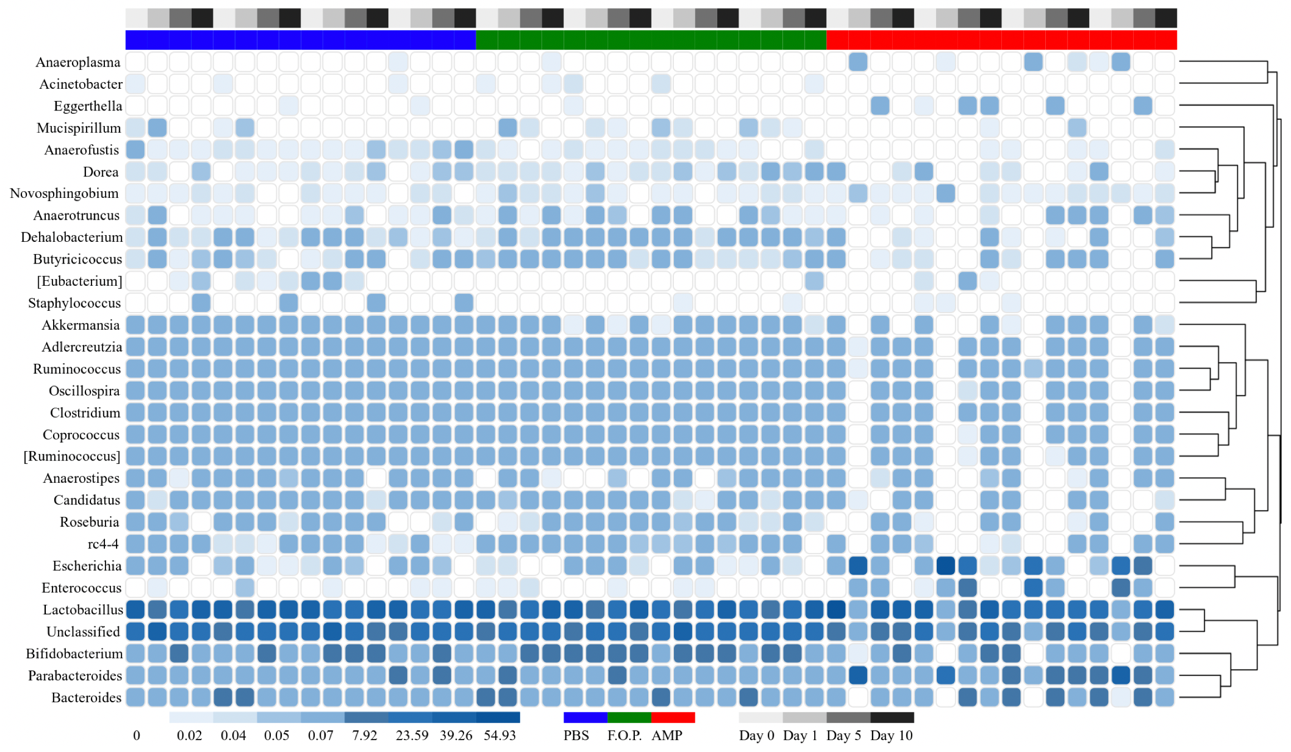


**Supplementary Figure A2.** Genus level heatmap (sorted by time). Displays the taxa identified in mouse fecal homogenates in chronological order of experiment days. Heatmaps were generated representing the relative abundance of taxa assigned to OTUs at the genus level. The columns were sorted by the treatment type, individual mouse, and day post-treatment.
